# Acute pancreatitis following asparaginase treatment in pediatric acute lymphoblastic leukemia with a heterozygous *SPINK1* c.194 + 2T>C intronic variant: a case report

**DOI:** 10.3389/fped.2024.1493362

**Published:** 2024-11-05

**Authors:** Hua Zhou, Jun Lu, Tao Wang, Xiaoyan Gu, Xueya Li, Jing Zhao

**Affiliations:** ^1^ Department of Hematology and Oncology, Affiliated Changzhou Children’s Hospital of Nantong University, Changzhou, Jiangsu, China; ^2^ Department of Hematology, Children’s Hospital of Soochow University, Suzhou, Jiangsu, China; ^3^ Zhejiang Key Laboratory of Digital Technology in Medical Diagnostics, Dian Diagnostics Group Co., Ltd., Hangzhou, Zhejiang, China; ^4^ Nanjing D.A. Medical Laboratory, Nanjing, Jiangsu, China; ^5^ Department of Pediatrics, Affiliated Changzhou Children’s Hospital of Nantong University, Changzhou, Jiangsu, China

**Keywords:** pancreatitis, asparaginase, acute lymphoblastic leukemia, *SPINK1*, c.194 + 2T>C, case report

## Abstract

**Background:**

Asparaginase is a critical component of chemotherapy for pediatric acute lymphoblastic leukemia (ALL), but its use is often complicated by asparaginase-associated pancreatitis (AAP). Genetic predispositions, such as variants in the *SPINK1* gene, have been linked to an increased risk of pancreatitis. However, the role of genetic factors in relation to asparaginase treatment remains incompletely understood, partly because mutations in pancreatitis-causing genes are rarely found in pediatric ALL.

**Case description:**

A four-year and three-month-old Chinese girl was admitted to our hospital due to fever for half a day, with no history of significant prior medical history. Initial blood tests revealed hematological abnormalities, including leukopenia, anemia, and thrombocytosis. Bone marrow aspiration identified 81.5% blast cells with B-lymphocyte morphology and immunophenotype, leading to a diagnosis of B-cell acute lymphoblastic leukemia (B-ALL). The patient began treatment under the CCCG-ALL-2015 protocol, which included PEG-asparaginase (PEG-asp). On day 10 of induction, she developed AAP, which was primarily characterized by severe epigastric pain and elevated serum amylase. Despite effective symptom management with analgesics and anti-inflammatory therapy, AAP recurred following administration of L-asparaginase (L-asp). Genetic analysis revealed a heterozygous *SPINK1* c.194 + 2T>C variant (rs148954387), a well-known pathogenic variant associated with increased susceptibility to pancreatitis. Sanger sequencing confirmed that the *SPINK1* variant was inherited from her asymptomatic mother. The patient's AAP was managed conservatively, and an asparaginase-free regimen ultimately achieved complete remission without recurrence of pancreatitis.

**Conclusions:**

The identification of the *SPINK1* c.194 + 2T>C variant, which is recognized as pathogenic, provides valuable information for understanding the heightened risk of AAP in our pediatric ALL patient. Our case underscores the potential role of genetic predisposition in the development of AAP and highlights the importance of considering genetic screening prior to asparaginase therapy in pediatric ALL patients to identify those at increased risk.

## 1 Introduction

Acute pancreatitis (AP) is characterized by the abrupt onset of pancreatic inflammation that arises when digestive enzymes activate within the pancreas itself, causing the organ to begin self-digestion (1–3). As a highly heterogeneous disease, AP presents a wide range of clinical manifestations, ranging from mild discomfort to more severe signs such as severe abdominal pain, nausea, and vomiting (1–3). Despite a survival rate about 95%, severe AP continues to be a major cause of death for those hospitalized patients with gastrointestinal diseases (1, 4, 5).

While both children and adults can develop AP, it is less common in children, with about 3–13 cases per 100,000 children per year, compared to 5–60 cases per 100,000 adults per year (6–8). In adults, gallstones and alcohol abuse are the predominant causes of AP, whereas in children, the etiology is diverse and often multifactorial (1, 2, 8). Common triggers in pediatric cases include medications, genetic factors, metabolic disorders, and infections (8). The intricate nature of AP, combined with the potential for severe complications and the necessity to address underlying causes, makes this condition particularly challenging to treat and prone to recurrence in children. It has been reported that nearly a third of children who experience AP will have at least one additional episode (9). Therefore, effective management of AP, especially in children, requires a tailored approach that considers the unique aspects of each patient.

AP is a significant and potentially serious complication in pediatric patients with acute lymphoblastic leukemia (ALL) undergoing chemotherapy, particularly when asparaginase such as L-asparaginase (L-asp) or PEG-asparaginase (PEG-asp), a pegylated form of L-asp, is included in the treatment regimen (10–12). Asparaginase catalyzes the hydrolysis of the amino acid asparagine into aspartic acid and ammonia. By depleting asparagine, asparaginase inhibits protein synthesis in leukemic cells, leading to their death. Although asparaginase is a critical component in the treatment of ALL, it comes with a range of potential adverse effects (13). Notably, asparaginase can cause the premature activation of pancreatic digestive enzymes within the pancreas (14, 15). This results in autodigestion of pancreatic tissue and ultimately asparaginase-associated pancreatitis (AAP), a distinct type of AP marked by the similar clinical signs of AP but with a specific etiology related to asparaginase therapy (10). The incidence of AAP varies, but it is estimated to occur in approximately 2%–18% of ALL patients, with a higher occurrence in older children and adults (10–12, 16, 17). AAP not only complicates pediatric ALL treatment but also increases the risk of mortality, necessitating vigilant monitoring and early intervention strategies.

What is noteworthy is that genetic factors also play a significant role in predisposing individuals to pancreatitis, with mutations in the *PRSS1* (protease serine 1), *SPINK1* (serine peptidase inhibitor Kazal type 1), *CFTR* (cystic fibrosis transmembrane conductance regulator), *CTRC* (chymotrypsin C), *TRPV6* (transient receptor potential cation channel subfamily V member 6), and *CPA1* (carboxypeptidase A1) genes being predominant contributors (18, 19). Recent machine learning studies by Nielsen et al. also identified several single nucleotide polymorphisms (SNPs) as potential risk factors for AAP, including rs10273639 (*PRSS1-PRSS2*), rs10436957 (*CTRC*), rs13228878 (*PRSS1-PRSS2*), rs1505495 (*GALNTL6*), and rs4655107 (*EPHB2*) (20). However, genetic screening for pancreatitis-causing genes in ALL patients is not currently standard practice, which limits our understanding of the impact of specific genetic variants on the development of AAP in pediatric ALL receiving asparaginase therapy. In this case report, we present a very rare instance of a pediatric B-cell ALL (B-ALL) patient with a *SPINK1* NM 003122: c.194 + 2T>C heterozygous variant (rs148954387) and AAP, highlighting the intricate interplay between genetic predisposition and asparaginase therapy in AAP development. By examining this case, we underscore the importance of genetic screening before initiating asparaginase therapy in pediatric ALL to identify at-risk patients and tailor treatment approaches accordingly.

## 2 Case presentation

### 2.1 The diagnosis of B-ALL

In October 2020, a four-year and three-month-old Chinese girl was admitted to the Affiliated Changzhou Children's Hospital of Nantong University due to fever for half a day. Her parents reported that the patient's maximum temperature reached 39.8°C at home, which could be temporarily relieved by physical cooling and ibuprofen. Transient chest pain and one episode of nonprojectile vomiting occurred during the fever, but there were no cold symptoms such as chills, cough, or a stuffy nose. Her parents were non-consanguineous, and her mother was gravida 1, para 1 (G1P1). The patient was born via cesarean section at term, with a birth weight of 3,100 g and a length of 50 cm. After birth, the patient had normal growth and development without significant medical history. A family history of hereditary, cancerous, and contagious diseases, as well as any epidemiologic connection to other COVID-19 cases or impacted regions, was denied by her parents.

On admission, the patient's temperature was 36.4°C, pulse rate was 108 beats/min, respiration rate was 24 breaths/min, and blood pressure was measured at 120/75 mmHg. Physical examination revealed enlarged tonsils and hyperemia of the pharynx. All other general and neurological examinations showed no abnormalities. Blood laboratory test revealed that the white blood cells count was 1.1 × 10^9^/L (normal range, 4–10 × 10^9^/L), monocytes count was 0.04 × 10^9^/L (normal range, 0.12–0.8 × 10^9^/L), and neutrophils count was 0.01 × 10^9^/L (normal range, 2–7.7 × 10^9^/L), all of which were remarkably reduced. The red blood cells count was 3.15 × 10^12^/L (normal range, 3.5–5.5 × 10^12^/L) and hemoglobin was 89 g/L (normal range, 110–160 g/L), both of which were also reduced. In addition, the platelet count showed an increase to 510 × 10^9^/L (normal range, 100–300 × 10^9^/L), and the C-reactive protein level was elevated to 30.03 mg/L (normal range, 0–10 mg/ml).

**Figure 1:**
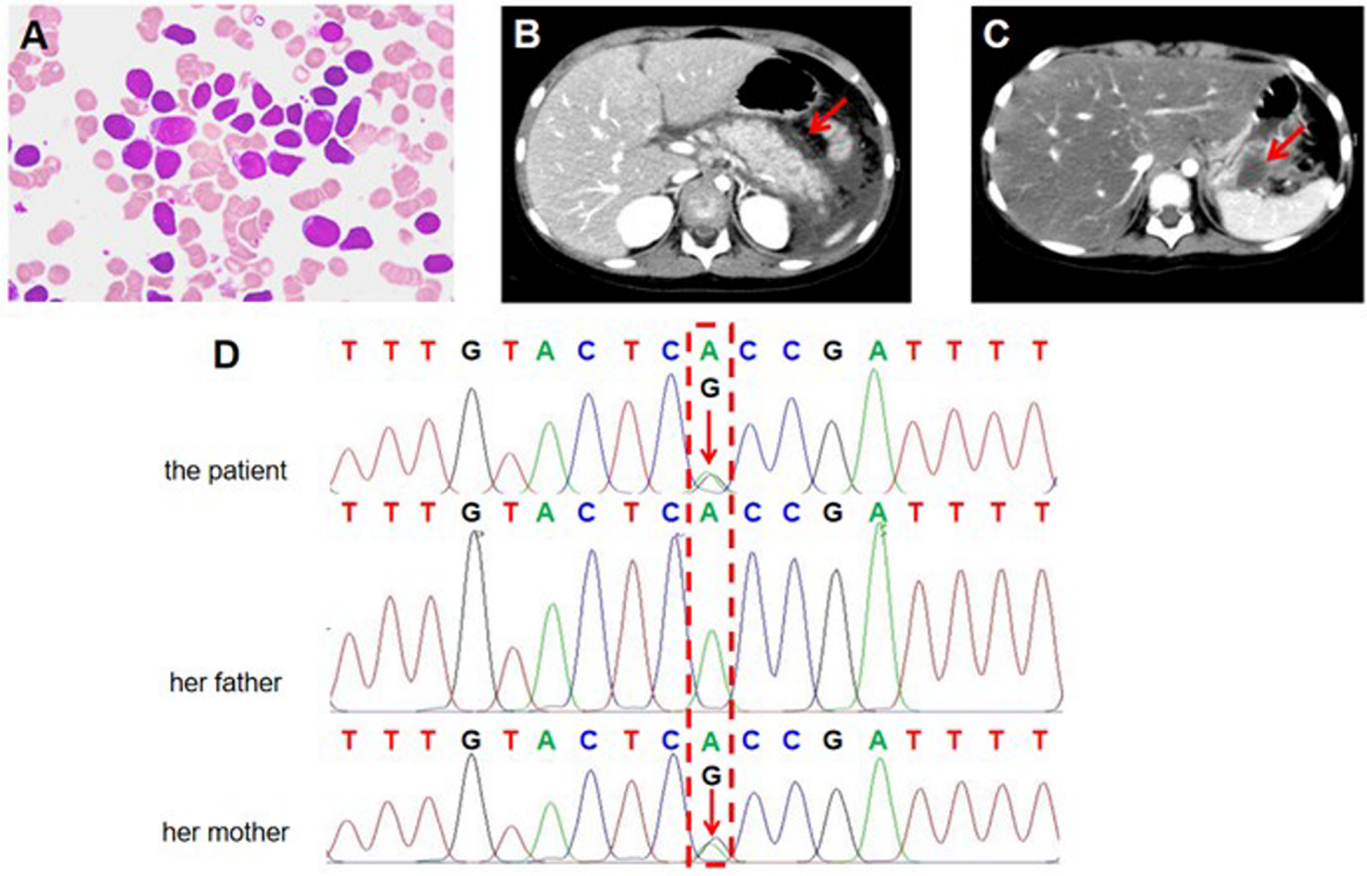
Clinical and genetic analysis of the investigated family. **(A)** Representative image of a bone marrow (BM) aspirate stained with Wright-Giemsa stain from the patient with B-cell acute lymphoblastic leukemia (B-ALL). The image shows a predominance of lymphoblasts, characterized by large, immature cells with scant cytoplasm, round to irregular nuclei, fine chromatin, and prominent nucleoli. **(B,C)** Contrast-enhanced axial abdominal CT scan of the pediatric patient showing features consistent with acute pancreatitis (AP). **(B)** The first episode of AP. The red arrow indicates that the pancreas is enlarged with diffuse peripancreatic fat stranding and peri-pancreatic fluid accumulation. The surrounding organs, including the liver and kidneys, appear unremarkable. **(C)** The second episode of AP. The red arrow indicates pancreatic pseudocyst. **(D)** Sanger sequencing (reverse strands) analysis of the *SPINK1* c.194 + 2T>C variant in the investigated family. The results show the B-ALL patient and her mother carries a heterozygous *SPINK1* c.194 + 2T>C variant. The patient's father has normal *SPINK1* gene. The red arrow indicates the identified variant position.

To investigate the cause of abnormalities in blood cell counts, we further performed bone marrow (BM) aspiration, which showed hyperplastic BM with 81.5% of blast cells exhibiting lymphoid morphology. The blasts were generally large with a high nucleus-to-cytoplasm ratio, finely dispersed chromatin, and prominent nucleoli (Figure 1A). All blasts were negative for myeloperoxidase (MPO) staining. Multiparameter flow cytometry of BM aspirate detected 89.6% blasts and showed an immunophenotype that was positive for CD10, CD19, CD34, CD38, and HLA-DR, and negative for CD2, CD7, CD13, CD14, CD15, CD20, CD33, and CD117, corresponding to B-ALL features. G-band analysis of BM metaphase cells was further performed to analyze chromosomal abnormalities. The karyotypes of ten analyzed cells were 46,XX[6]/54,XX,dup(1)(q21q31),+4,+6,+10,+14,+17,+18,+21,+21[1]/55,idem,+8[1]/56,idem,+6,+8[2] (Figure 1B). Although common fusion genes typically found in leukemia were not detected in this patient, further transcriptome analysis using bulk RNA sequencing (RNA-seq) detected a rare *RNF213-SLC26A11* fusion gene. Taken together, the patient eventually was diagnosed with B-ALL.

### 2.2 B-ALL treatment and the development of AAP

According to Chinese Children's Cancer Group study ALL-2015 (CCCG-ALL-2015) protocol, the patient was classified as low-risk ALL and treated with standard induction regimen, which included vincristine, daunorubicin, PEG-asp, and dexamethasone. On day 10, the patient developed vomiting and severe epigastric pain. Serum amylase was elevated to 508.0 U/L, which was more than three times the upper limit of normal. Abdominal computed tomography (CT) showed pancreas edema, suggesting the development of AAP (Figure 1B). Therefore, the patient fasted for one week and was treated with analgesic therapy (octreotide) and anti-inflammatory therapy (ulinastatin) for ten days, which significantly relieved her epigastric pain. On day 19, the minimal residual disease (MRD) was 1.2 × 10^−2^ by flow cytometry. Therefore, the patient continued to take the same regimen as re-induction, albeit PEG-asp was replaced by L-asp. Notably, symptoms of pancreatitis reoccurred immediately after L-asp administration. On abdominal CT scan, pancreatic pseudocyst was detected (Figure 1C). Thus, both PEG-asp and L-asp were discontinued. After symptomatic treatment such as fasting, fluid intake, octreotide, and ulinastatin, the patient's symptoms were gradually improved. On day 46, end-of-induction BM aspiration revealed 0% blasts by flow cytometry MRD. Following the CCCG-ALL-2015 protocol, the patient underwent consolidation therapy with high-dose methotrexate (MTX) and maintenance therapy with low-dose MTX and mercaptopurine, leading to complete remission of ALL.

### 2.3 Genetic analysis

The patient's parents were both healthy with no history of pancreatitis or hematological diseases. To accurately determine the cause of our patient's AAP, we collected her peripheral blood genomic DNA and performed whole-exome sequencing (WES) analysis using standard protocols. Data quality assurance, bioinformatics analysis, and interpretation were conducted following the American College of Medical Genetics and Genomics (ACMG) guidelines and established approaches. In our analysis of the exome data, we specifically examined genes associated with hematological and metabolic diseases, as well as those related to pancreatitis, including *PRSS1*, *SPINK1*, *CFTR*, *CTRC*, *TRPV6*, and *CPA1*. After filtering common (allele frequency of >5%) and non-coding variants, we found a heterozygous variant NM_003122.2: c.194 + 2T>C in intron 3 of *SPINK1* gene (rs148954387), which may contribute to the development of AAP. Sanger sequencing confirmed the presence of *SPINK1* c.194 + 2T>C intronic variant in the patient (Figure 1D). Further analysis revealed that the patient's healthy mother was also heterozygous for *SPINK1* c.194 + 2T>C, while the father carried the normal *SPINK1* gene (Figure 1D). According to the ACMG guidelines, *SPINK1* c.194 + 2T>C is defined as a pathogenic variant (PVS1, PS1). Based on clinical and genetic evidence, we conclude that the *SPINK1* c.194 + 2T>C variant increases the susceptibility to AAP during asparaginase treatment in our B-ALL patient.

## 3 Discussion

AAP is a significant and common adverse effect observed during asparaginase-based therapy for ALL, particularly in pediatric patients. Although the precise mechanism of AAP is multifactorial and not fully elucidated, it likely involves a combination of inappropriate pancreatic enzymes activation, immune responses, and metabolic disturbances that collectively increase the risk of pancreatic inflammation and injury (21). One of the mechanisms by which asparaginase induces pancreatic injury is through toxic calcium (Ca^2+^) signaling, which eventually leads to necrosis of pancreatic acinar cells (22). For accurate diagnosis of AAP in pediatric ALL patients, a thorough evaluation is essential. This includes a detailed assessment of clinical history, symptoms, laboratory tests, and imaging studies (1–3). For our patient, the key evidence for diagnosing AAP included abdominal pain that occurred rapidly following asparaginase administration, elevated serum amylase levels (more than three times the upper limit of normal), and appearance of pancreatic edema on CT scan.

Although genetic factors may influence the development of AAP, routine screening for mutations in pancreatitis-related genes is not commonly performed before treatment in pediatric ALL. As a result, the specific role of these gene mutations in AAP occurrence in pediatric ALL remains poorly documented and is considered rare. In this report, we identified the *SPINK1* c.194 + 2T>C variant (rs148954387) as key pathogenic factor of the AAP in a B-ALL patient. To the best of our knowledge, this is the first report identifying this *SPINK1* variant in pediatric ALL. The *SPINK1* gene encodes a single-chain polypeptide known as pancreatic secretory trypsin inhibitor (PSTI), a protein approximately 6.3 kDa in size that plays a crucial role in preventing the activation of trypsinogen, the inactive precursor of trypsin, within the pancreas (18, 19, 23). PSTI is estimated to inhibit around 20% of the pancreas's total potential trypsin activity. Mutations in the *SPINK1* gene are widely perceived to reduce the activity of PSTI, allowing trypsinogen to be prematurely converted into trypsin within the pancreatic tissue (24). This excessive proteolytic activity may lead to autodigestion of the pancreas and initiates inflammation, contributing to the pathogenesis of pancreatitis. Although PSTI is primarily known for its ability to inhibit trypsin, its broader biological roles and interactions with other pancreatic proteins and enzymes remain incompletely understood, necessitating further research to clarify these functions and their implications for pancreatitis.

In fact, the *SPINK1* c.194 + 2T>C variant (rs148954387), also known as IVS3 + 2T>C, is one of the most common genetic variants associated with pancreatitis, particularly in East-Asian populations (18). According gnomAD V4.1.0, the variant allele was found at a total frequency of 0.0001137 (25). c.194 + 2T>C variant occurs in the splice donor site of intron 3 of the *SPINK1* gene. The pathogenic impact of this intronic variant is well-documented (26–28). The *SPINK1* c.194 + 2T>C heterozygous variant has been independently demonstrated in multiple studies to disrupt pre-mRNA splicing, which significantly reduces the expression of full-length *SPINK1* mRNA (26–28). Using pancreatitis mouse model, a recent study by Liu et al. demonstrated that the heterozygous *Spink1* c.194 + 2T>C mutation promotes the development of pancreatitis by elevating interleukin-33 levels, inducing M2 macrophage polarization, and activating pancreatic stellate cells (29). However, like other pathogenic variants of pancreatitis, the *SPINK1* c.194 + 2T>C variant is also found in healthy individuals, suggesting that additional factors such as other genetic variants or lifestyle influences likely play a significant role in disease expression (18). Indeed, our B-ALL patient had no symptoms of pancreatitis prior to this hospitalization but developed symptoms only after receiving asparaginase. Notably, her mother, who also carries the *SPINK1* c.194 + 2T>C heterozygous variant, has not exhibited noticeable symptoms of pancreatitis in the past. Nevertheless, given that *SPINK1* c.194 + 2T>C variant increases susceptibility to pancreatitis, the patient's mother has been advised to maintain a healthy lifestyle and to seek medical attention if any symptoms associated with pancreatitis develop.

A definitive consensus on managing ALL in patients with *SPINK1* or other gene mutations that predispose them to AAP has not yet been established. In general, the management of AAP is a delicate balance between addressing the acute episode, preventing further complications, and ensuring that leukemia treatment remains effective, all while minimizing the risk of recurrent pancreatitis. Most patients with AAP require hospitalization, particularly if the episode is severe. When managing our patient's AAP, a typical supportive care approach includes fasting to rest the pancreas, intravenous fluids to maintain hydration, anti-inflammatory treatment to prevent infection, and pain management. This conservative approach is effective for most patients, although more severe cases might require additional interventions like nutritional support via enteral feeding or even surgical procedures if complications arise. Frequently, AAP leads to truncation of asparaginase therapy, which can increase the risk of leukemia relapse, making the reintroduction of asparaginase a critical consideration. Notably, studies indicate that reintroducing asparaginase after an initial episode of AAP carries a 40%–50% risk of a second pancreatitis episode (10, 11). Therefore, the decision to reintroduce asparaginase must be guided by a careful risk-benefit analysis. We must acknowledge that reintroducing asparaginase in our patient, primarily guided by the MRD level on day 19, without fully understanding her genetic background, carries significant risks. Fortunately, conservative treatment effectively controlled the symptoms of pancreatitis and prevent recurrence, and the asparaginase-free chemotherapy achieved an MRD-negative state without causing complications. As of the time of writing, there has been no recurrence of pancreatitis in the past four years.

This case report has several limitations that should be considered. First, as a single case study, the findings may not be generalizable to all pediatric ALL patients undergoing asparaginase therapy. The identification of the *SPINK1* c.194 + 2T>C variant and its association with AAP in this patient highlights the potential importance of genetic screening, but the rarity of such findings limits the applicability of this approach in routine clinical practice. Additionally, although we performed comprehensive genetic analysis, other potential genetic, environmental, or treatment-related factors contributing to AAP were not fully explored. The absence of long-term follow-up data beyond four years also limits our understanding of the patient's risk of pancreatitis recurrence or other potential complications. Lastly, the decision to reintroduce asparaginase based on MRD levels, while achieving leukemia remission, poses a significant risk, which may not be acceptable in all clinical contexts. Further research is needed to establish standardized protocols for managing asparaginase therapy in patients with genetic predispositions to AAP.

## 4 Conclusions

To summarize, the identification of the *SPINK1* c.194 + 2T>C variant, which is recognized as pathogenic, provides valuable information for understanding the heightened risk of AAP in our pediatric ALL patient. Our case underscores the potential role of genetic predisposition in the development of AAP and highlights the importance of considering genetic screening prior to asparaginase therapy in pediatric ALL patients to identify those at increased risk.

## Data Availability

The original contributions presented in the study are included in the article/Supplementary Material, further inquiries can be directed to the corresponding author.
